# Toward Zero Waste Mining: Circular Economy of Copper Slags

**DOI:** 10.1002/gch2.202500392

**Published:** 2025-10-26

**Authors:** Aleksandar Mitrašinović, Željko Kamberović, Dawei Yu

**Affiliations:** ^1^ Institute of Technical Sciences of the Serbian Academy of Sciences and Arts Knez Mihailova 35 Belgrade 11000 Serbia; ^2^ Faculty of Technology and Metallurgy University of Belgrade Karnegijeva 4 Belgrade 11120 Serbia; ^3^ School of Metallurgy and Environment Central South University Changsha 410083 China; ^4^ National and Regional Joint Engineering Research Center of Nonferrous Metal Resource Recycling Changsha 410083 China

**Keywords:** circular economy, copper slag, geotechnical applications, green mining, value‐added products, zero waste

## Abstract

Only 8.6% of the global economy follows the principles of the circular economy. Mining and metal extraction processes generate over 90% of global waste, with slags being the major contributor to waste accumulation. Copper slags mostly consist of stable compounds that are safe for the environment, the main concern being the leaching of heavy metals into the ground. The market for using waste copper slags to produce value‐added products is established in the last century and grown to an 800 million dollar market in 2024, with a predicted compound annual growth rate of 5.5% in the following years. However, only 15% of the generated copper slags worldwide are used for commercial purposes. In order to attain complete reusability of copper slags, industrial techniques must be adopted to eliminate threats from heavy metals and other possibly harmful elements. Additionally, it is essential to establish a market that can sustainably handle all quantities of generated slag. This review highlights the properties that make copper slags suitable for producing value‐added products. With the rapid increase in population and urbanization, there is a potential to utilize copper slags for a‐large‐scale construction purposes such as earthworks and buildings. The demand for geotechnical applications and construction materials constantly increases and can absorb the entire quantity of copper slags generated. If this happens, it would mean an achievement of 100% reusability of copper slags in compliance with circular economy principles.

## Graphical abstract

Mining and metal extraction processes generate over 90% of global waste. With the rapid increase in population and urbanization, the demand for geotechnical applications and construction materials constantly increases and can absorb the entire quantity of slags generated. If this happens, it would mean an achievement of 100% reusability of slags in compliance with circular economy principles.

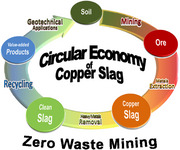

## 1 Introduction

The three guiding principles of the circular economy are to reduce waste and pollution, reuse materials and products to the fullest extent possible, and restore the environment.^[^ ^1^, ^2^, ^3^ ^]^ Considering the global waste, surpassing 90% is generated by mining and metal extraction processes^[^ ^4^, ^5^, ^6^, ^7^ ^]^ but a mere 8.6% of the global economy operates according to the circular economy principles.^[^ ^8^, ^9^, ^10^ ^]^ The waste produced during mining and metal extraction consists of sturdy slags, aqueous mine tailings and residues from hydrometallurgical processes, and dusty materials generated by both the processing of ferrous and non‐ferrous metals. Each of these materials has the potential to cause environmental damage. Addressing the circularity gap is essential for preventing additional environmental degradation and social inequality.^[^ ^11^ ^]^ If a solution is found to convert mining and metal extraction waste into value‐added products, a reduction of ≈90% in globally disposed wastes in landfills could be achieved.^[^ ^12^, ^13^ ^]^

Slags, which are byproducts formed during the pyrometallurgical extraction of metals from ores, have long been regarded as waste. One example of such a material is copper slag, a byproduct generated during the smelting and converting of copper. Smelting forms two phases: a copper‐rich matte and a slag consisting of oxides.^[^ ^14^ ^]^ Despite technological advances, due to the inherited constitution of the ores, producing one tonne of copper generates at least twice as many tonnes of slag.^[^ ^15^, ^16^, ^17^ ^]^ As a result, the global slag generation from annual copper production is estimated to be around 24.6 million tonnes.^[^ ^18^ ^]^ The known approaches to managing these slags involve recycling, recovering valuable metals, engendering value‐added products,^[^ ^19^, ^20^, ^21^, ^22^ ^]^ or, in most cases, disposal into the environment.

Copper slag (CS) is a dense, hard, and low‐absorption material. It is composed mainly of iron oxide and silicon dioxide. It has a low loss on ignition, low chloride and sulfate contents, and without the risk of alkali‐silica reaction.^[^ ^23^, ^24^ ^]^ When slowly cooled to ambient temperature, it forms a crystalline structure similar to crushed rock typically used in the construction industry as coarse aggregates.^[^ ^25^ ^]^ At the same time, rapidly cooled slag produces a granular sand‐like, mostly amorphous material.^[^ ^26^ ^]^ It is used as an abrasive material and a fine concrete aggregate since slag's impact and crushing values, and friction angle are similar to or better than natural aggregate.^[^ ^27^, ^28^, ^29^ ^]^ However, the slag quantities accumulated over the centuries and further generated each year are much greater than the demand for abrasive and aggregate products.

In Europe, since the Roman Empire, ferrous slags have been used for geotechnical applications,^[^ ^30^, ^31^ ^]^ while in the United States, these slags have been used as engineering fill and road pavement materials since the 19^th^ century.^[^ ^32^, ^33^ ^]^ Some researchers reported using copper slags in ground granulated form as a chemical stabilizer or granular copper tailings for natural soil replacement.^[^ ^34^, ^35^ ^]^ The primary concern with using copper slags in geotechnical applications is their potential environmental impact because of the leaching of heavy metals and potentially toxic elements,^[^ ^36^ ^]^ which may occur when copper slag comes into contact with water (rainwater, groundwater, surface water from flooding, or streams and rivers).

The rapid increase in population and urbanization in developed and emerging economies is leading to a decrease in suitable land for infrastructure development.^[^ ^37^, ^38^ ^]^ The claim of slag heaps and landfills containing such materials for urbanization coincides with the potential to use these materials for construction purposes, including earthworks, pavements, and buildings. This increase in demand for geotechnical applications and construction materials is particularly relevant for countries like China and the United States, conceding that copper slags are readily available in these countries.^[^ ^39^ ^]^

This paper provides the origin and justification of the vast amounts of copper slag accumulation. Emphasis is given to the copper slags' physicochemical and mechanical properties, which make them suitable materials for geotechnical applications and other value‐added products. Known examples of the implemented methods for slag reclamation and revalorization are given. Examinations of the positive socioeconomic impacts on communities near the substantial slagheaps are highlighted to support the adoption of circular economy principles.

The review protocol methodology involved several steps for systematically collecting, sorting, and removing data from the literature. Various search terms and strings were entered into the ScienceDirect database. The primary search phrase used was “copper slag”, combined with terms such as “recovery”, “value added”, “circular economy”, “geotechnical application”, and “socioeconomic impact”, utilizing the “AND” operator to refine the query. To focus specifically on copper slags, exclusion terms like “iron” and “blast furnace” were applied. Once the relevant literature was gathered, the selection process prioritized more recent publications. Finally, the data were condensed to include only the referenced publications.

## 2 Copper Extraction and Slag Origin

The copper‐extraction smelting charge mainly consists of sulfides and oxides of iron and copper. Additionally, it includes oxides like Al_2_O_3_, CaO, MgO, and SiO_2_. The amounts of iron, copper, sulfur, and oxygen primarily control the smelting system's chemistry. Another crucial factor is the oxidation/reduction potential of the gases used to heat and chemically react with various charge components.

The primary purpose of smelting is to melt and recombine the ore charge into a homogeneous matte comprised of metals in metallic or sulfide form while simultaneously forming slag from iron and silicon oxides. In the smelting of copper, most of the targeted material is bound in chalcopyrite (CuFeS_2_), which decomposes during smelting through the following chemical reactions:

(1) $$2\mathrm{CuFe}{S_{2}}_{\left(\mathrm{ore}\right)}\;+\;4O_{2}\to Cu_{2}S_{\left(\mathrm{matte}\right)}\;+\;2\mathrm{Fe}O_{\left(\mathrm{slag}\right)}\;+\;3S{O_{2}}_{\left(\mathrm{gas}\right)}$$

while the generalized chemical reaction equation for both smelting and converting is:

(2) $$\mathrm{CuFe}{S_{2}}_{\left(\mathrm{ore}\right)}+\left(4+x\right)/2O_{2}\to Cu_{\left(\mathrm{mate}\right)}\;+\;\mathrm{Fe}{O_{x}}_{\left(\mathrm{slag}\right)}\;+\;2S{O_{2}}_{\left(\mathrm{gas}\right)}$$

The predominantly covalent bonding in copper‐sulfur compounds differs from the ionic bonding in the silicate components of the slag. These differences in chemical bonding are the primary driving force toward forming the matte phase, where these sulfidic compounds preserve their distinct bonding characteristic of the matte phase. Metals that remained in slag tend to coalesce with the entrapped copper sulfides that did not coalesce into the matte. As a result, the copper slag's final microstructure contains copper‐sulfide prills and other metals dispersed in a matrix of iron and silicon oxides. Although concentrations of these metals in the slag are small, they pose a significant challenge when considering the circular economy of copper slags and the need to remove heavy and potentially toxic elements from these slags.

Also, some copper in charge is bound in CuO, CuSO_4_, CuO/CuSO_4_, or CuO/Fe_2_O_3_ compounds. During smelting, these compounds also react to form either CuS or Cu_2_S. Because of their high sulfur pressure, CuS and FeS_2_ are unstable at high temperatures. As a result, if present, they also decompose and generate Cu_2_S and FeS.^[^ ^40^, ^41^ ^]^ The sulfidization of copper bound in oxides can be best formalized by the following equation:^[^ ^42^, ^43^, ^44^ ^]^

(3) $$\mathrm{Fe}S_{\left(l\right)}\;+\;Cu_{2}O_{\left(l;\;\mathrm{slag}\right)}\;=\;\mathrm{Fe}O_{\left(l;\;\mathrm{slag}\right)}\;+\;Cu_{2}S_{\left(l\right)},$$

The corresponding equilibrium constant for Equation (3) is as follows, where all species are in a liquid state (*a* corresponds to activities):

(4) $$K_{E}=\frac{a_{cu2}s_{\left(l\right)}\times a_{Fe}o_{\left(l,slag\right)}}{a_{cu2}o_{\left(l,slag\right)}\times a_{Fe}s_{\left(l\right)}}$$

**Figure 1:**
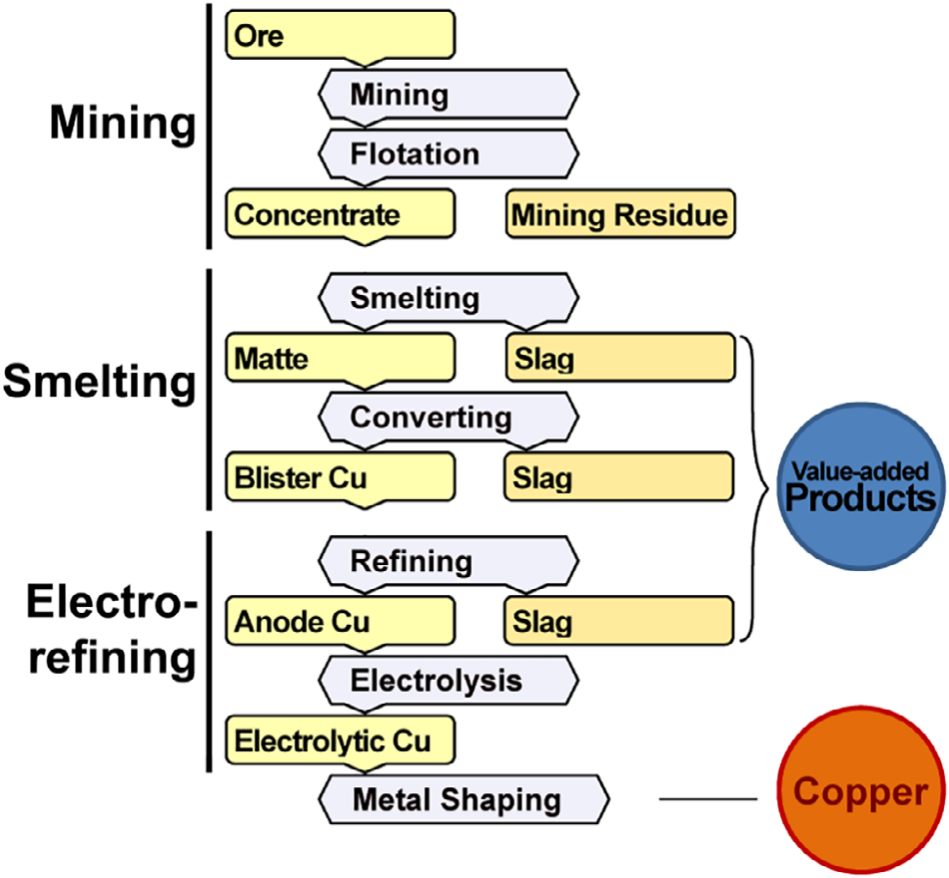
Flow‐sheet of the copper extraction process, slag origin, and generated value. The mining, smelting, and electrorefining of copper are distinct steps in the process of gaining copper from ore. Mining extracts the ore, while smelting uses high heat in furnaces to separate copper from impurities, producing impure blister copper. Electrorefining utilizes electric current to dissolve impure copper and consequently deposit pure copper onto cathode sheets.

The equilibrium constant (*K_E_*) is ≈10^4^ at the preferred smelting temperature, around 1200 °C. This theoretical consideration implies that, because of the high value of *K_E_*, Cu_2_O can be completely sulfidised to Cu_2_S by FeS. Equations (3) and (4) may also be used for kinetics considerations regarding recovering copper from converter slags, since converter slags are typically returned to the smelting furnace. **Figure** **1** provides the origin of the copper slag throughout the extraction process.

The slow cooling of liquid slag forms a compact and rigid crystalline substance, whereas rapid solidification through pouring molten slag into water yields amorphous granulated slag. Variations in the chemical composition of slag differ because of different ore properties, extraction technologies, and applied process parameters. Regardless of the origin of the slag, copper content is limited to amounts below one percent, except in rare cases of slags formed by ancient ore‐extraction practices. Significant constituents are iron oxides, silica, alumina, and calcium oxide. The following is the usual composition of copper slag. Iron: 30–40%, SiO_2_: 35–40%, Al_2_O_3_: 0–10%, CaO: 0–10%, Cu: 0.5–2%.

## 3 Physicochemical Properties

The appearance of an air‐cooled copper slag is black and glassy. The specific density of copper slag varies depending on its iron content, ranging from 2.8 to 3.8 kg cm^−3^.^[^ ^45^, ^46^ ^]^ The density of copper slag is higher than that of conventional aggregate. Copper slag has a shallow absorption capacity of 0.13% on average. Granulated copper slag is more porous, which results in lower specific gravity and higher absorption capacity than the air‐cooled copper slag. The granulated copper slag comprises regularly shaped, angular particles, primarily in the size range of 0.075–4.75 mm.^[^ ^47^, ^48^, ^49^ ^]^

Microscopic observations from various sites have shown that iron oxides, silica, alumina, lime, and magnesia make up 95% or more of the total microstructure in copper slags. The X‐ray diffraction patterns mostly identify 2FeO:SiO_2_ (fayalite), Fe_3_O_4_ (magnetite), and SiO_2_ (quartz) as the main phases present in the slag.^[^ ^50^ ^]^ As metals are most stable in oxide and silicate forms, construction materials produced from copper slag have the slightest possibility of corroding.

The copper slags, if heated, will remain unchanged until temperatures are around 350 °C.^[^ ^51^ ^]^ After this point, the oxidation of Fe_3_O_4_ to form Fe_2_O_3_ begins. At temperatures around 550 °C, transformations of magnetite to maghemite while at 700 °C, maghemite further transforms to hematite. If copper and iron sulfides are present in copper slag, they will oxidize at around 850 °C.^[^ ^52^ ^]^ Hence, based on temperature alone, there would be no structural changes in copper slag materials below 350 °C. If necessary, further immobilization of the copper slags can be carried out relatively simply by increasing the temperature to 1000 °C, which is still below the melting point of copper slag. This would result in the decomposition of the less stable components and the formation of oxidic compounds, mainly unaffected by atmospheric conditions.

Even without further immobilization, considering low concentrations of the heavy and potentially toxic metals, the properties of most air‐cooled and granulated copper slags make them a suitable material for use as an aggregate because of their favorable mechanical properties. This slag has a sharp, angular shape, resulting in a high friction angle. However, the slag can be vitreous or glassy, which is unfavorable for material used on pavement surfaces because of a negative impact on skid resistance.

## 4 Mechanical Properties

Because of the large quantities of slag generated, a significant amount will continue to be disposed of in landfills. To ensure that the geological configuration is not significantly altered and the natural habitat remains intact, the water fluctuation characteristics, the quickness of consolidation settlement, and compressibility properties of the disposed slags must be similar to those of the natural soil.

**Figure 2:**
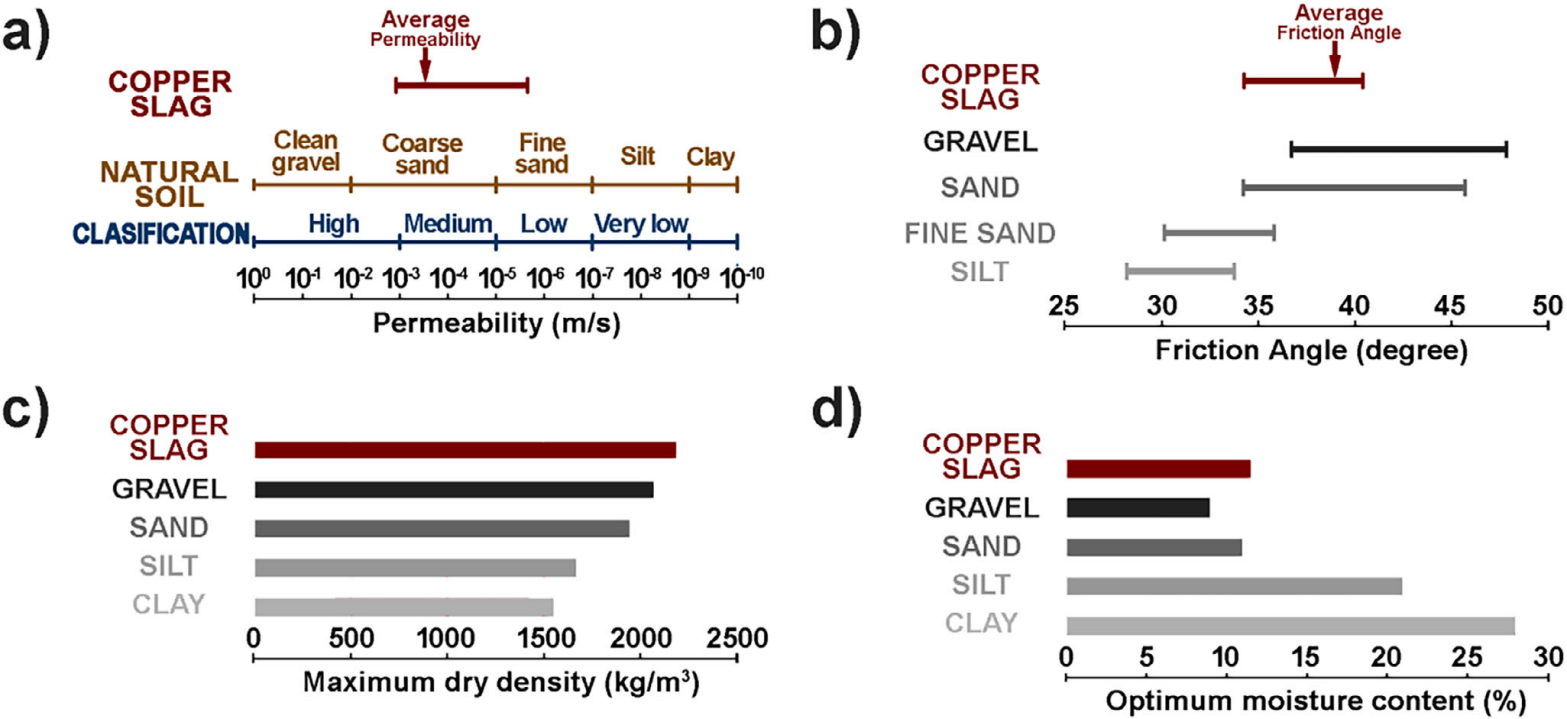
The comparison of copper slag properties with typical materials constituting the natural soil. a) permeability, b) friction angle, c) maximum dry density, and d) optimum moisture content.

The ease with which water can flow through the pores of the soil, known as permeability, depends on particle size distribution, porosity ratio, pore interconnection, and soil saturation capacity.^[^ ^53^ ^]^ **Figure** **2**a provides permeability values for various materials that make up natural soils. The lowest reported permeability values for copper slags are around 1 × 10^−6^ m s^−1^, while most measurements fall between 1 × 10^−3^ and 1 × 10^−4^ m s^−1^, with the average value being 3 × 10^−4^ m s^−1^.^[^ ^54^ ^]^ Compared to natural components of the soil, many researchers noted that these values are equivalent to coarse sand.^[^ ^55^, ^56^ ^]^ Various mixtures of natural soil containing 10–40% copper slag have a permeability that closely resembles that of natural sand.^[^ ^57^, ^58^ ^]^ Therefore, copper slags may be an excellent substitute for sand, particularly in layered soils, which can reduce groundwater absorption, in the foundations of hydraulic structures that can prevent internal erosion, or in areas with poor drainage where they can be used as coarse material.

When copper slags are disposed of in landfills, they mix with the natural soil, leading to consolidation settlement, which changes soil stability. The speed and size of consolidation settlement depend on the soil's permeability and compressibility. Soils with a fine‐grained structure, such as silts and clays, consolidate slowly due to their low permeability. However, in soils with a coarse‐grained structure, like gravels and sands, consolidation happens quickly due to their high permeability. Since copper slags and sands have similar permeability, they would have similar consolidation settlement values.

Compressibility is a term used to express how soil particles arrange themselves into a stable structure once the water is drained from the soil mass. For materials with no cohesion, like sand, compressibility depends on the particle shape, grading, and applied external force. The reports on the compressibility of copper slag and sand mixtures over several decades are inconclusive due to differences in the origin, particle sizes, and shapes of the studied materials and the different standard test methods employed. However, angular fine sand is more compressible than rounded fine sand. Since copper slag has a high angularity compared to any variation of natural sand with a similar particle size distribution, the copper slag is expected to have better compressibility.

Soil engineers consider shear strength to be crucial in assessing the soil‐bearing capacity of embankments, the stability of slopes, and the pressure exerted by soil on retaining walls. The shear strength of coarse granular soils, such as gravels and sands, is purely frictional, as no cohesion is present. The measurements of the friction angle by direct shear test of various copper slags, collected by Lambe and Whitman,^[^ ^59^ ^]^ are shown in Figure 2b, comparing them to typical natural soils. The higher friction angle of copper slags is expected since copper slag particles are more angular than natural sand, which provides a greater degree of particle interlocking to resist shear motion.

Permeability and compressibility properties contribute to the stability and load‐bearing capacity of copper slags, surpassing the requirements specified for embankment and fill construction set by the US Federal Highway Administration,^[^ ^60^, ^61^ ^]^ implying that these materials can sustain and more effectively transfer loads than some typically used materials (Figure 2c,d).

However, there may be risks to the environment due to the presence of heavy and potentially toxic elements.^[^ ^62^, ^63^ ^]^ It is sometimes necessary to extract these metals to ensure that the copper‐slag products are environmentally safe.^[^ ^64^ ^]^ These additional extraction procedures can significantly improve the environmental soundness of the value‐added copper‐slag products and assure safe usage in geotechnical applications.

## 5 Valuable Metals Recovery and Slag Cleaning

When slag undergoes treatment to remove heavy metals, the extracted metals become valuable.^[^ ^65^, ^66^ ^]^ Some copper slags, previously overlooked, contain significant concentrations of rare earth elements (REEs), offering a promising avenue for recovering these metals.^[^ ^67^, ^68^, ^69^ ^]^ The selection of a process for the recovery of valuable metals depends on various factors, such as the intended use of the slag, the level of intended waste minimization, the availability of reactants, economic feasibility, and the amount of targeted metals in slag. Pyrometallurgical and hydrometallurgical processes, flotation, sorting, and combinations of these techniques are typically used to recover valuable metals.^[^ ^70^ ^]^ According to the World Copper Smelter Survey, 37% of smelters used electric furnaces to recover metals, 26% used flotation, 6% did not perform additional slag cleaning, 5% used other technologies, and no information was available for 26% of the smelters.^[^ ^71^ ^]^

Flash/Continuous technology dominates copper extraction, accounting for 67%, followed by 16% copper gained by leaching ores,^[^ ^72^ ^]^ both generate enormous amounts of mining waste. In commercially utilized processes for recovering valuable metals from copper slags, typically, copper and nickel were recovered by flotation and leaching,^[^ ^73^, ^74^, ^75^, ^76^ ^]^ iron by carbothermic reduction and magnetic separation,^[^ ^77^ ^]^ and one facility recovered cobalt by reduction processes.^[^ ^78^, ^79^ ^]^

While pyrometallurgical processes have large industrial capacities, they face environmental impact and energy consumption disadvantages; in hydrometallurgical processes, the leaching of potentially valuable elements is a key research focus, but the low concentration of these elements often can satisfy economic incentives.^[^ ^80^, ^81^ ^]^ Thus, the future development of pyrometallurgical processes should focus on conserving energy and reducing emissions.^[^ ^82^ ^]^ Utilizing renewable resources, such as biomass, along with clean energy sources like hydrogen, demonstrates significant promise for the future.^[^ ^83^ ^]^

Historically, research and practices were driven by large mining companies concentrated on recovering valuable metals, often without regard for environmental impacts. However, as the principles of the circular economy gain wider acceptance, previous studies offer valuable data that can improve the quality of slags reused for value‐added products.^[^ ^84^ ^]^ Interestingly, the methods and techniques developed for recovering valuable metals are largely similar to those used to remove heavy and toxic elements.

### 5.1 Removal of Heavy Metals by Gravity Separation

Considering the energy and resources invested, gravity separation is the most efficacious method for removing heavy metals.^[^ ^85^ ^]^ Either toxic or valuable metals are typically much heavier than the oxides found in copper slag. Some heavy metals with lower thermal stability (i.e., Hg, Cd, As, and Pb) are prone to volatilization and may be evaporated at higher temperatures.^[^ ^86^ ^]^ These heavier components settle at the bottom when the slag is heated and kept above its melting point for a significant amount of time, while less dense iron and silicon oxides will remain in the upper part of the refractory vessel. Material formed at the bottom of the containing vessel is typically called matte. Matte formation by settling at temperatures above the slags’ melting point is widely used in various industries.^[^ ^87^ ^]^ This method is mainly conducted in large‐sized ladles without energy input or large‐capacity batch furnaces. Furnaces for slag cleaning are similar to a Peirce‐Smith Converter, which eases logistics and operational challenges. Recently, Isaksson et al.^[^ ^88^ ^]^ showed that in an industrial environment, with varying conditions, a 56% copper recovery can be achieved on an input copper content of 1.25 wt.%, where significantly increasing the settling time begins reducing the copper recovery, which matches Mitrašinović and Wolf^[^ ^89^ ^]^ work at the University of Toronto, where settling for longer than 2 h decreased recovery rates.

Separation related only to the difference in densities of the present species would not remove entirely unvented elements. Injection of a strong reducing agent, most often carbon, contributes to the chemical reactions where most metals are initially reduced from oxides and then settled in the matte.^[^ ^90^ ^]^

### 5.2 Removal of Heavy Metals by Carbon

Copper slag is commonly treated with carbon‐containing additives to retrieve iron and other metals, producing an iron‐rich alloy^[^ ^91^, ^92^ ^]^ while the remaining residues have little commercial value and are typically disposed of.^[^ ^93^, ^94^, ^95^ ^]^ Along with copper, copper‐rich slag heaps typically contain other valuable metals. Using carbothermic reduction in Kure (Turkey), from initial material containing 1.72% Co and 4.4 % Cu, Topkaya^[^ ^96^ ^]^ and Yücel et al.^[^ ^97^ ^]^ recovered 97.7% Co and 90.0% Cu when slag was treated at 1400 °C for 1 h. Recently, Seyrankaya and Canbazoǧlu^[^ ^98^ ^]^ achieved the extraction efficiencies of cobalt, copper, and zinc at 96.82, 92.85, and 93.44%, respectively, after subsequently leaching slag with H_2_SO_4_ for 1 h at 220 °C with oxygen partial pressure 0.7 MPa.

In a practical application, carbon‐containing materials are preferably reducing materials because of their price, availability, and ease of transport and storage. As a result of carbothermic reactions, carbon monoxide (CO) and carbon dioxide (CO_2_) are produced. Considering the amount of carbon used for carbothermic reduction in various industries, these gases are major contributors to greenhouse emissions. When using carbon‐containing additives, the optimal temperature for slag cleaning processes is high because reactions between iron and carbon or carbon oxides require an external heat supply due to their predominantly endothermic nature.

Regardless of serious drawbacks in using carbon‐containing compounds, due to the lack of alternative reducing agents available in large quantities at a low price, the carbothermic‐based processes, for some time, will keep a leading role in slag cleaning processes. Hence, one of the intermediate research goals, before new reducing material is found, is to adjust process parameters to enhance oxide reduction without significantly raising the temperature. This includes increasing carbon activity, reducing the size of the reducing agents, and enhancing stirring conditions throughout the process.^[^ ^99^ ^]^

### 5.3 Removal of Heavy Metals by Carbonless Additives

**Figure 3:**
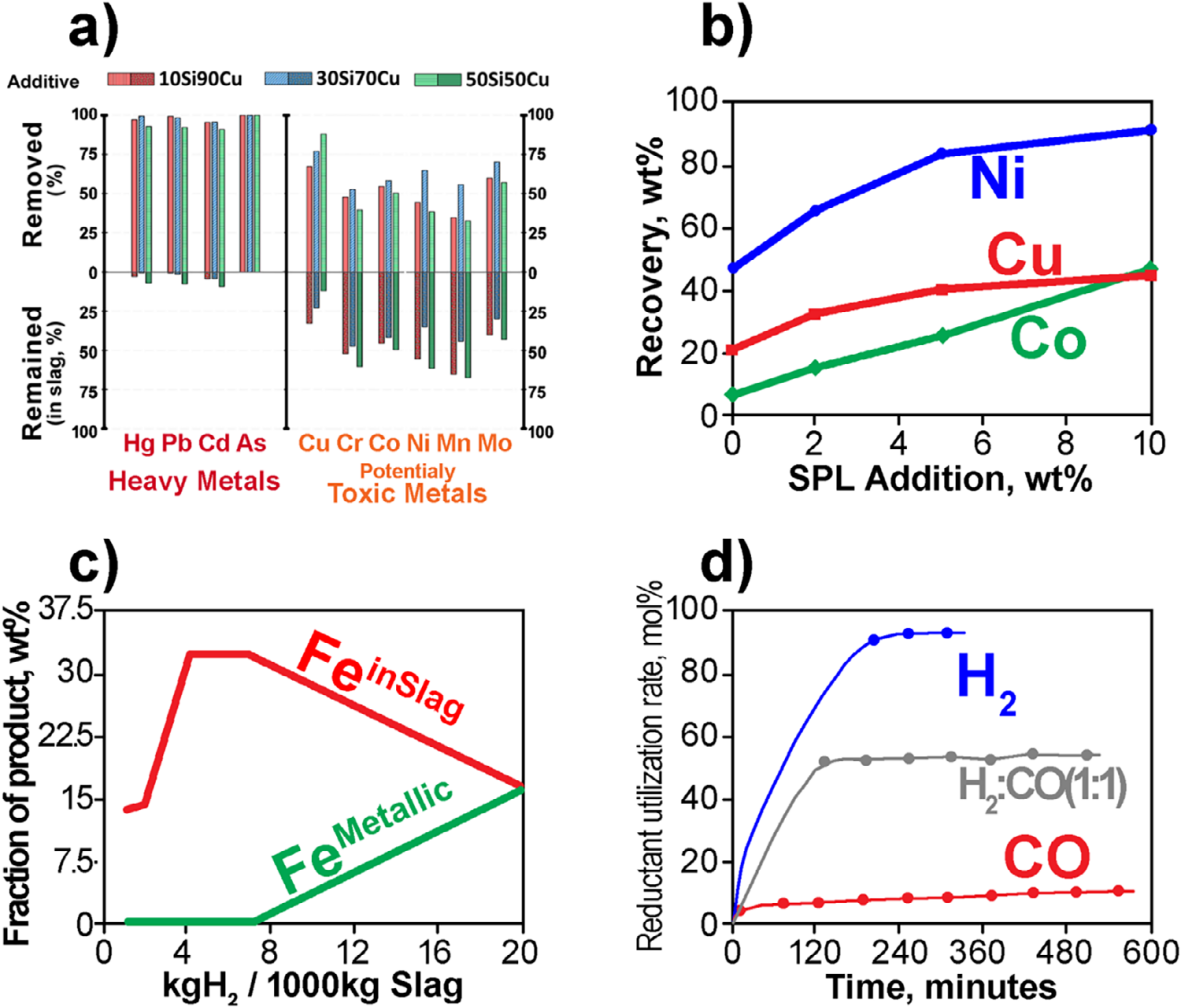
The reduction of heavy and potentially toxic metals from copper slag to acceptable levels through the application of carbonless reducing compounds, where all processes are scalable and applicable in an industrial environment. a) Removal of heavy and toxic metals using silicon‐copper intermetallic compounds that are byproduct of silicon refining process. Reproduced with permission.^[^ ^100^ ^]^ Copyright 2025, Elsevier. b) Metals recovery using fluoride values in spent potlining (SPL). Reproduced with permission.^[^ ^102^ ^]^ Copyright 2018, Springer Nature. c) Overall mass fraction of products reduced to the metallic form using hydrogen. OpenAccess Article.^[^ ^104^ ^]^ reative Commons Attribution 4.0 International License. and d) The rates of reductant utilization across various CO/H_2_ ratios. Reproduced with permission.^[^ ^105^ ^]^ Copyright 2021, Elsevier. According to the World Health Organization, heavy metals such as cadmium (Cd), lead (Pb), mercury (Hg), and metalloid arsenic (As) are systemic toxicants known to induce damage to living organisms, while cobalt (Co), copper (Cu), chromium (Cr), iron (Fe), magnesium (Mg), manganese (Mn), molybdenum (Mo), nickel (Ni), selenium (Se), and zinc (Zn) are important nutrients but at elevated concentrations can pose a potential toxicity risk to living organisms.

The reduction process involves various reducing agents and specific treatment methods that extraction companies often keep confidential. As a result, it is challenging to find a commercial slag cleaning facility that fully adheres to circular economy principles. However, laboratory research has identified alternatives to current industrial practices. **Figure** **3** illustrates the potential to reduce heavy and potentially toxic metals to acceptable levels by using different reducing substances during a slag settling process, where all of the presented processes are scalable and can be implemented in an industrial setting.

Based on work originated at the University of Toronto,^[^ ^50^ ^]^ Mitrašinović et al.^[^ ^100^ ^]^ kept the slag at a temperature of 1300 °C for 4 h, improving the metal recovery by mixing it with silicon‐copper compounds that are byproducts of the silicon refining process.^[^ ^101^ ^]^ The levels of six heavy metals decreased from 2400 ppm in the original slag to 41.7 ppm when using a 30 wt.%Si‐70 wt.%Cu compound (Figure 3a). Mixing the slag with additives with high silicon content made the oxygen concentrations in the resulting mattes ≈50 times lower than those in the initial slag. These methods can help reduce the hazards linked to heavy and potentially harmful metals in copper slag disposal while facilitating the recovery of valuable metals and generating additional revenue.

Yu and Chattopadhyay^[^ ^102^ ^]^ developed a numerical model and experimentally confirmed an innovative approach to enhance copper recovery and copper slag's operational efficiency by adding fluoride values found in spent potlining (SPL) from the aluminum industry. The fluorides and sodium‐containing compounds in SPL lowered the slag viscosity, leading to a quicker settling rate of matte droplets. The findings revealed that adding ≈3 wt.%SPL to the molten copper converter slag could achieve a 90% Cu recovery rate. In this process, the SPL could be detoxified by converting cyanides into harmless nitrogen gas and neutralizing the fluorides in a much‐diluted form in the ferro‐silicate slag, ensuring safe disposal into the environment.

### 5.4 Removal of Heavy Metals by Hydrogen

The development of technologies targeted at nearly totally eliminating CO_2_ emissions is an initiative that requires the creation of innovative, carbon‐free technologies for decreasing undesired materials from the discarded slags. An environmentally friendly alternative for the conventional removal of toxic materials from the copper slag, in compliance with the circular economy principles, is hydrogen reduction.^[^ ^103^ ^]^

Attah‐Kyie et al.^[^ ^104^ ^]^ treated non‐ferrous slags with a high iron content using scalable pyrometallurgical methods to recover the valuable metals and transform the remainder into a benign slag. The experiments were conducted at temperatures between 1200 and 1300 °C using hydrogen and nitrogen to form the reducing gas atmosphere. They obtained more than 12% of the overall mass of the slag in metallic form (Figure 3c).

Zhang et al.^[^ ^105^ ^]^ conducted comprehensive kinetics studies in comparing the reduction efficiencies of the carbon monoxide and hydrogen gases. Figure 3d displays the rates of reductant utilization across various CO/H_2_ ratios, noticing a sharp increase reaching almost the maximum reductant utilization rate when hydrogen is used. The kinetics of the metallization reactions were zero order when the CO/H_2_ mole ratios were high and changed to first order reactions when the ratios decreased to around one. They recognized that in cases where the inlet gas is a blend of CO, H_2_, and Ar, a reaction may occur between H_2_O and CO alongside deoxygenation. This additional reaction could improve the reductant utilization rates and efficiency of the reduction processes.

### 5.5 Removal of Heavy Metals by Traditional Metallurgical Techniques

Like slag heaps, tailings accumulate near smelting operations containing between 0.2% and 0.4% copper.^[^ ^106^ ^]^ In such cases, hydrometallurgical methods could be economically feasible if other metals are extracted along with copper. Sigedin et al.^[^ ^107^ ^]^ optimized the flotation process used at the Almalyk copper smelter to process slags and Cu/Mo ores and presented satisfactory outcomes. In the 1980s, Oprea and Murgulescu^[^ ^108^ ^]^ suggested the process for recovery of valuable metals from a solution containing 0.30–0.65% Cu, leading to profitable outcomes elsewhere.^[^ ^109^, ^110^, ^111^ ^]^ They used concentrated sulphuric acid at 80/90 °C and obtained a solution with 0.14 g L^−1^ Cu, from which copper could be extracted through well‐known processes. The US Environmental Protection Agency reported significant recoveries of valuable metals in the White Pine Copper Division of the Copper Range Co., an industrial facility built specifically for the recovery of metals from the reverberatory furnace slags.^[^ ^112^ ^]^

In the 1990s, attempts were made to utilize the carbothermic reduction process for commercial metal recoveries. Acma et al.^[^ ^113^ ^]^ reduced fayalitic slag containing ≈0.8% Cu and 0.4% Co using coke/graphite in a DC arc furnace. The result was a predominantly iron‐containing alloy with ≈3.8% Cu, 3.3% Co, and 2.1% S. This alloy was then treated with hot sulfuric acid, separating copper and other impurities in the residue. Subsequently, the leached liquor was treated with H_2_S to precipitate cobalt as sulfide, and finally, the remaining liquor, mainly FeSO_4_, underwent goethite precipitation, dehydration, controlled reduction, and reoxidation to obtain Fe_2_O_3_ susceptible to magnetic separation. The direct reduction process with coal demonstrated its viability in recovering various metals from converted copper slag that can be used to produce alloys like Fe‐Cu‐C, which are used in steel production.^[^ ^114^ ^]^

### 5.6 Commercialized Processes for Valuable Metals Recovery

As high‐grade ores become scarcer, the potential to retrieve basic and economically valuable metals such as iron, copper, cobalt, or nickel from copper slags is recently gaining traction.

Pilot plant tests at Mintek (South Africa) showed that 98% nickel and 80% cobalt recoveries can be achieved.^[^ ^115^ ^]^ Lately, another study used an electric arc furnace to partially reduce FeO from the slag, obtaining a metallic phase with low containment of the toxic elements.^[^ ^116^ ^]^ Near the Nkana slag heaps in Zambia, which contain up to 0.66% cobalt in copper slag, Mopani Copper Mines Plc produces cobalt‐bearing alloys with Co content ranging from 5% to 14%.^[^ ^117^, ^118^ ^]^

Considering the emphasis on the economic value of the commercialized enterprises for valuable metals recovery, it is equally important to underline the need to comply with circular economy principles and environmentally concerned guidelines, where slags should find a value‐added purpose rather than being disposed of in landfills wherever possible.

## 6 Value‐Added Products

**Figure 4:**
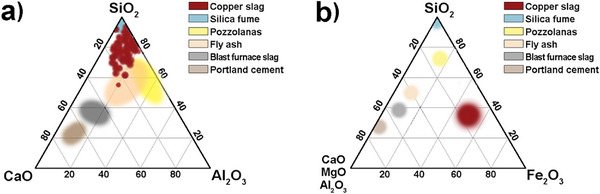
Comparisons of the chemical compositions of established cementitious materials. a) CaO‐Al_2_O_3_‐SiO_2_ ternary diagram and b) (CaO+MgO+Al_2_O_3_)‐SiO_2_‐Fe_2_O_3_ system. [Reproduced with permissions.^[^ ^119^ ^]^ Copyright 2011, Elsevier.

Copper slags are primarily composed of iron oxides (Fe_x_O_y_) and silica (SiO_2_), along with smaller amounts of alumina (Al_2_O_3_), calcium oxide (CaO), and magnesium oxide. **Figure** **4**a compares the chemical composition of copper slags with commonly observed compositions of Portland cement, ground granulated blast furnace slag, fly ash, silica fume, and natural pozzolanas. Due to its high silica content, copper slags’ chemical composition is similar to fly ash and silica fume. However, its Fe_x_O_y_ content sets it apart from other cementitious materials (Figure 4b). High iron content is useful as a fluxing agent in ceramics production by creating robust and durable products that are highly resistant to damage. Also, the high concentration of Fe_2_O_3_ increases the slag's overall density, which may reduce transportation and storage costs.

Copper slags have advantageous physicochemical properties for various applications, which, together with the reduction of heavy metal contamination, have resulted in a broader range of applications in various industries.^[^ ^120^ ^]^ These applications include abrasive tools, pavement, concrete, cutting tools, tiles, glass, roofing granules, cement, and asphalt concrete aggregate. **Table** **1** outlines the value‐added products derived from copper slags.

**Table 1:** The representative reports regarding value‐added products derived from copper slags.

Value‐added product	Usage	Refs.
Abrasive/cutting tools	grinding	[121, 122, 123]
	blasting	[124]
	polishing metals and alloys	[125]
	machining steel	[126]
	component in ceramic binders	[127, 128]
Tiles and glass	filler	[129]
	tiles	[130, 131, 132]
	colored glasses	[133, 134]
Pavement	hot‐mix paving	[135, 136]
Concrete	admixtures in concrete and mortars	[137]
	filler	[54]
	Portland cement replacement	[138, 139]
	fine aggregate	[140]
Technical applications	backfilling	[141, 142]
	railway ballast and sleepers	[143, 144, 145]

## 7 Geotechnical Applications

**Figure 5:**
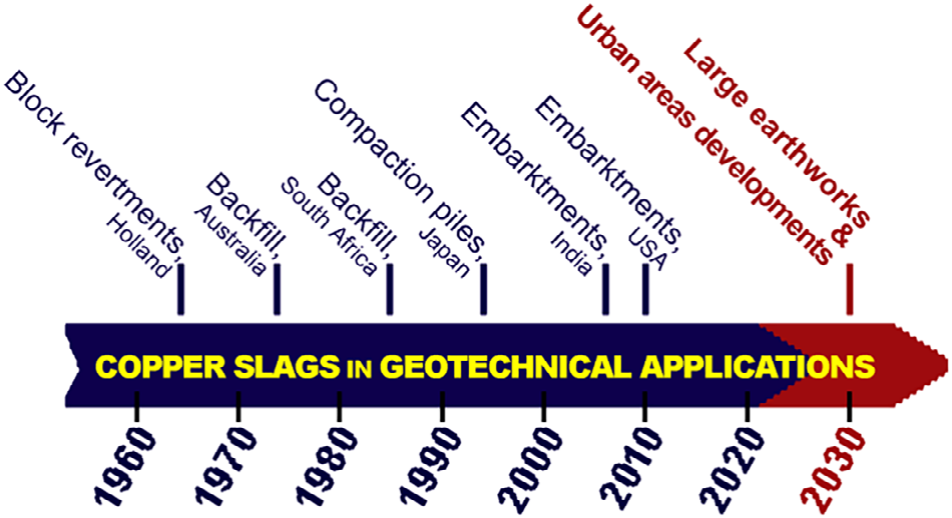
Timeline of the utilization of the copper slags for geotechnical applications.

Since the 1960s, copper slag has been utilized as a natural substitute for sand in various construction projects, such as revetments, sand compaction piles, and embankments^[^ ^146^, ^147^ ^]^ (**Figure** **5**). It can also be used as a binder for mine backfill.^[^ ^148^, ^149^ ^]^ Field applications of copper slags have reported no significant problems. For geotechnical applications, guidelines suggested in the ASTM(2011) unified soil classification system describe copper slag's particle size and distribution, liquid limit, and plasticity index.

When copper slag is combined with silt, clay, or fly ash, the mixtures' plasticity index decreases linearly as the copper slag content increases. The Proctor test ascertained copper slag's highest possible dry density at 2.2 g m^−3^ and the ideal moisture content at 11.5%. Mixing copper slag with various soils or ashes can adjust the dry density and moisture content values. Copper slag's compaction characteristics are better than those of natural sand.

The permeability and rate of consolidation for copper slag and sand are very similar. However, slag's permeability may be slightly reduced when used with sand due to better particle packing of the resulting mixture. Moreover, copper slag is more compressible than rounded natural sand of similar grading due to its angularity.

The average friction angle of copper slags is around 39°, which is close to the middle value for well‐graded sand. When various soils, sands, and ashes are mixed, the friction angle increases with increasing copper slag content. When copper slag is used as backfill material for retaining‐wall structures, the active earth pressure coefficient decreases linearly with increasing copper slag content. The average decreasing rate is 4% for every 10% increase in copper slag content in the mixture.

Testing has confirmed that the leaching of elements from copper slag, when used in geotechnical applications and exposed to pH environments ranging from 3 to 8, has concentrations well below the regulatory levels set by the US Environmental Protection Agency. Even when a mixture of copper slag with other construction materials is tested according to set standards, the original chemical composition of the leached material remains similar.

The geotechnical properties of copper slags are comparable to or surpass those of natural sand. Monitoring results of practical field applications alongside laboratory studies can accelerate future improvement of engineering requirements while better preserving the environment. Considering amounts, using copper slag in geotechnical applications in earthworks, revetment, and embankment, or ecological concretes would mean 100% reuse in complete alignment with the circular economy principles. Finding applications for reusing the entire mining waste excess would reduce more than 90% of waste not incorporated into the circular economy loop.

## 8 Environmental Considerations

Mining plays a vital role in the economies of many societies. However, along with mining waste, metallurgical operations generate tailings and slags as byproducts, which can have harmful environmental effects.^[^ ^150^, ^151^ ^]^ In the past, little attention was paid to properly handling and disposing of mining and metallurgical waste, as the primary objective was maximizing profits.^[^ ^152^ ^]^

The Basel Convention of the United Nations, which regulates the transboundary movement of hazardous wastes and their disposal, has classified copper slag as non‐hazardous since 1996.^[^ ^153^, ^154^ ^]^ The decision was based on examining slag samples from Canada, Chile, and the United States, which evaluated 32 years of data from 10 different countries on the leaching concentrations of heavy metals from copper slag,^[^ ^155^, ^156^ ^]^ where all heavy metal concentrations were below the permitted limits set by international organizations.

It is important to carefully evaluate the evidence, such as statements, bills, and references that demonstrate the safety of copper slags concerning their impact on the environment. Although specific locations may meet the permitted limits of heavy metals and toxic elements, specific areas may contain significantly higher concentrations due to changes in extraction technologies and dumping practices. Therefore, safe practices require that all types of slags intended for use as value‐added products of geotechnical applications undergo regular leaching tests, regardless of their overall concentration of heavy metals and potentially toxic elements.

## 9 Socioeconomic Effects

The United Nations Basel Convention on the Transboundary Movement of Hazardous Wastes and Their Disposal removed copper slags from the list of hazardous materials because of low concentrations of heavy and potentially toxic metals. For the same reason, in most cases, earlier research focused on attempts to recover metals from the copper slags for monetary gains deemed unsuccessful. When considering the reuse of slags, it is important to consider the positive socioeconomic effects for the entire society, rather than focusing only on isolated profitable assessments.

In most cases, the regions that accumulate the most slag are also the least developed countries, which further deepens socioeconomic concerns in these regions. Ahmed et al.^[^ ^157^ ^]^ estimated that adopting practices of reusing copper slag as a secondary source may provide over 10000 jobs per average mining site.

Using copper slag as a secondary source for the extraction of metals provides social security for a particular region for a long time and generates tens of thousands of new jobs in environmentally friendly industries instead of traditional mining/extraction operations. Copper slags could be considered an essential resource for the environment, economy, and society rather than just a waste product. Standards‐compliant mines and extraction facilities preserve the environment by recovering heavy metals, while they may have commercial worth from these operations. Less reactive or inert slags, depleted of heavy metals, can be used in the construction industry for various purposes.^[^ ^158^ ^]^

Research conducted in the past has revealed that high‐grade ores have been exhausted over time, and waste materials, including copper slags, may contain minerals that can be extracted. Therefore, it is essential to analyze these slags to determine their mineral and chemical composition, which can help identify potential methods for mineral extraction. Iron ore is considered valuable if it contains at least 35% Fe, a requirement met by all copper slags. Iron is a crucial element for economic growth, and copper is also an essential metal, with its current market price being the highest ever.

## 10 Perspectives

In 2020, the Global Copper Slag Market had a valuation of US$580.73 million.^[^ ^159^ ^]^ The market is predicted to expand to US$803.24 million by 2027^[^ ^160^ ^]^ and exceed a Compound Annual Growth Rate (CAGR) of 5.5%.^[^ ^161^ ^]^ With tighter regulations, the mining and metallurgy industries will face harsher challenges in solid waste disposal, which motivates civil and environmental engineers to develop new processes and technologies to utilize waste generated from various industries. Therefore, there has been a significant increase in scientific publications analyzing copper slags in recent years. Increased scientific community attention further helps promote sustainable development while enabling mining and metallurgical enterprises to earn additional revenue from waste disposal.

Cement and concrete producers are currently the largest re‐users of copper slags. They mix copper slag with limestone powder and dust to produce cement, mortar, and/or concrete. They are also beginning to replace sand in concrete and mortar, resulting in more durable, less permeable, and lower water‐absorption rate products, making them ideal for large‐scale concrete applications.^[^ ^162^ ^]^ Copper slags are also an eco‐friendly option for open‐space blasting used in the automotive industry. Simultaneously, with the increased demand for copper slag by large‐scale industries, the primary metals producers will have additional economic benefits from this practice, particularly in regions where large amounts of copper slag are produced.

Administrations across different countries are taking active steps to promote effective waste conversion into value‐added products. Together with the governmental incentives, the market for copper slags is rising for other reasons, such as increasing environmental concerns, depletion of natural reserves, rising prices of raw metal ores, or increasing urbanization and industrialization rates in developing countries. The emergence of new technologies also contributes to better practices in turning waste into value. Along with North America, the most significant copper slag market growth is in the Asia‐Pacific region. The future increased demand for copper slags in these regions is driven by increasing urbanization, industrialization, and many metal extraction facilities that generate copper slags. Along with market incentives, government initiatives, and anticipated large construction projects for residential and commercial purposes further encourage the growth of the copper slag market in these regions.

Key factors contributing to the increased reuse of copper slags are the rising demand for green buildings, government efforts to support effective waste management, growing public awareness of environmental sustainability and waste management, higher demand for alternatives to conventional construction materials, and the significantly decreasing availability of waste landfill space in the future.

**Figure 6:**
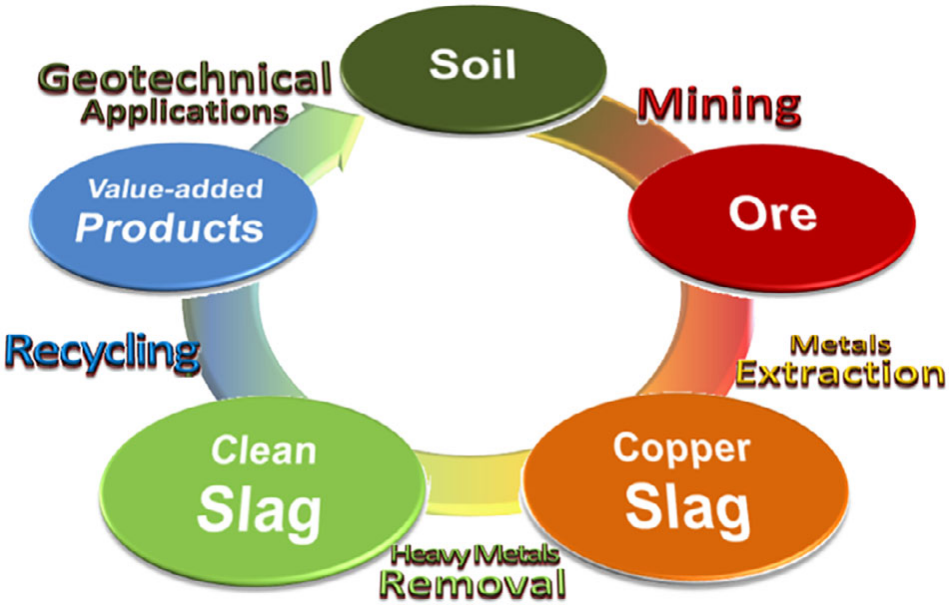
Circular economy of copper slag.

There is no doubt that the reuse of copper slag, by volume, will grow over time. Economic indices, such as CAGR (Compound Annual Growth Rate), predict significant returns from investments focusing on reusing copper slags. Government incentives for reusing copper slags are growing, including in developing countries, while control and regulations are getting tighter regarding the amounts of allowed heavy metals. Social awareness and acceptance regarding improving the environment already exist. The reuse of copper slags is a rare example where all socioeconomic factors are positive, indicating a future increase in slag reuse. However, the pace of copper slag's transition toward 100% compliance with circular economy principles, as depicted in **Figure** **6**, relies heavily on educating the public about the potential benefits of using slag rather than viewing it as waste.

## 11 Conclusions

Copper slags produce a variety of value‐added products because of their favorable physicomechanical and chemical characteristics. These products include cement, fill, ballast, abrasive‐cutting tools, aggregate, roofing granules, glass, and tiles. Compared to similar conventional materials, the materials created from copper slag have superior mechanical properties and, in many cases, perform better. Using copper slag in this way can reduce disposal costs and environmental challenges, and improve production economics. Additionally, some copper slags contain valuable metals that can be extracted through different metallurgical methods. Recovering these valuable metals improves the production economics of the value‐added products and makes copper slags suitable for replacing natural soil.

Copper slags are already utilized in building revetments, sand compaction piles, and embankments. They have excellent engineering properties and can replace natural sand in geotechnical applications or as a standalone material in earthworks, where water absorption is not an issue. Considering the amounts of the existing and future generated slags and the amount of materials required in geotechnical applications, adopting practices of using copper slags for earthworks modifications of the urban areas and areas affected by human activity could be a significant milestone in mining waste management toward achieving 100% compliance with the circular economy principles.

## Conflict of Interest

The authors declare no conflict of interest.

## Author Contributions

A.M.M. did writing of the original draft, visualization, methodology, investigation, formal analysis, and conceptualization. Ž.K. did writing of the original draft, conceptualization, and supervision. D.Y. did writing of the original draft, visualization, and validation.
